# A case report of young-onset type 2 diabetes patient with severe metabolic dysregulation carrying an *LRP6* variant

**DOI:** 10.3389/fendo.2026.1887705

**Published:** 2026-08-27

**Authors:** Dan Xiang, Li Yuan, Shuaiju Liao, Yangtian Wang

**Affiliations:** Department of Endocrinology, Taikang Xianlin Drum Tower Hospital, Affiliated Hospital of Medical School, Nanjing University, Nanjing, China

**Keywords:** case report, hypertriglyceridemia, insulin resistance, LRP6, metabolic dysregulation, type 2 diabetes, variant, young-onset

## Abstract

**Background:**

The global epidemic of type 2 diabetes mellitus (T2D), along with its rising prevalence among younger populations, raises substantial public health concerns; severe hypertriglyceridemia (sHTG) is a major risk factor for acute pancreatitis. Insulin resistance in T2D often coincides with dyslipidemia, contributing to lipotoxicity, and insulin-resistant states may be linked to specific genetic variants.

**Case presentation:**

This report presents a 27-year-old man with poor glycemic control, recurrent dizziness, extreme hypertriglyceridemia (TG > 37.87 mmol/L), insulin resistance, pancreatic fat infiltration, and early-stage diabetic nephropathy. Despite a family history of T2D, his clinical presentation far exceeded that of typical T2D. High-throughput sequencing revealed a heterozygous *LRP6* variant (c.3107A>G, p.Asn1036Ser).

**Outcome:**

Multidisciplinary treatment—incorporating acute-phase plasma exchange, ω-3 fatty acid ethyl ester 90, inclisiran, subcutaneous insulin aspart pump therapy, metformin, insulin icodec, finerenone, and Huangkui capsules—led to improvements in the patient’s metabolic indicators.

**Significance:**

This case report describes a patient with type 2 diabetes mellitus presenting with extreme metabolic disorders, in whom a variant in the *LRP6* gene was detected. Although the variant co-occurs with the observed phenotype, its pathogenicity cannot currently be established in the absence of functional validation. This case emphasizes that genetic variant screening should be considered in patients with unexplained severe metabolic disturbances, particularly those with early-onset disease. This case also provides a foundation for future functional studies and genetic confirmation.

## Introduction

Type 2 diabetes mellitus (T2D) is a globally prevalent metabolic disorder whose incidence continues to rise, with an alarming trend toward younger age at onset (1). Although advances in T2D management have been made in recent years, including the introduction of novel glucose-lowering agents, sustained glycemic control remains difficult to achieve (2). Severe hypertriglyceridemia (sHTG), defined as triglyceride (TG) concentrations ≥ 5.7 mmol/L, represents a major risk factor for acute pancreatitis. The underlying pathophysiology often involves genetic defects in proteins that regulate lipoprotein lipase (LPL) activity (3). In patients with T2D, insulin resistance is a core defect; beyond driving hyperglycemia, it frequently co-occurs with dyslipidemia, causing lipotoxicity that further exacerbates pancreatic β-cell dysfunction and systemic metabolic dysregulation (4). Of note, insulin resistance has been associated with specific genetic variants affecting insulin signal transduction or adipose tissue distribution, which can manifest as extreme metabolic phenotypes including severe hypertriglyceridemia, early-stage diabetic nephropathy, and pancreatic fat infiltration (5).

This case report describes a 27-year-old man with poorly controlled glycemia, recurrent dizziness, extreme hypertriglyceridemia (TG > 37.87 mmol/L), and insulin resistance. The distinctiveness of this case lies in its early onset and the severity of metabolic dysregulation, complicated by a history of pancreatitis and evidence of early-stage diabetic nephropathy (urinary microalbumin-to-creatinine ratio of 601.98 mg/g). Despite a clear family history of type 2 diabetes, his clinical presentation far exceeded that of typical type 2 diabetes. Imaging studies revealed pancreatic fat infiltration, consistent with non-alcoholic fatty pancreatic disease (NAFPD). This form of ectopic fat deposition is closely linked to metabolic syndrome and insulin resistance and can impair both endocrine and exocrine pancreatic function (6). The coexistence of preserved yet relatively insufficient C-peptide levels, severe hyperglycemia, markedly elevated proinsulin, and a high C-peptide-based insulin resistance index collectively indicates insulin resistance and β-cell stress.

This case report’s principal value lies in its presentation of a young patient with type 2 diabetes, a severe metabolic phenotype, and a variant of uncertain significance (VUS) in *LRP6*. LRP6 encodes low-density lipoprotein receptor-related protein 6, a critical co-receptor for the Wnt/β-catenin signaling pathway that is essential for insulin sensitivity, lipid metabolism, and pancreatic development and function. By integrating the clinical phenotype (severe hypertriglyceridemia, insulin resistance, pancreatic steatosis, and early-stage nephropathy), biochemical parameters (extreme dyslipidemia and hyperproinsulinemia), and genetic findings, this case offers a valuable clinical illustration for exploring the link between LRP6 variants and severe metabolic disorders in humans. Moreover, the multidisciplinary treatment strategy used—acute-phase plasma exchange; lipid lowering with ω-3 fatty acid ethyl ester 90 and inclisiran; combined metformin and insulin therapy to control hyperglycemia and address insulin resistance; and finerenone plus Huangkui capsules (a Chinese herbal formulation used to reduce proteinuria in diabetic kidney disease) to lower urinary protein and protect kidney function—provides a practical reference for managing such complex presentations. Therefore, the importance of early systematic genetic evaluation in young patients with extreme metabolic abnormalities is underscored by this case report, although individualized treatment was not directly guided by the identified LRP6 variant. Not only is the clinical understanding of the phenotype of type 2 diabetes patients harboring an LRP6 variant of VUS and concomitant severe metabolic disorders enhanced by this report, but the interplay between insulin resistance and lipotoxicity and potential therapeutic strategies are also elucidated.

## Case presentation

### Patient information

The patient was a 27-year-old male admitted due to recurrent dizziness and poorly controlled blood glucose. He had a 5-year history of diabetes mellitus. Over the preceding 2 years, he had been managed with a regimen consisting of subcutaneous injection of 20 units of insulin glargine at bedtime, combined with subcutaneous injection of 20 units of insulin aspart before each meal. He also reported a 2-year history of pancreatitis and denied any history of smoking or alcohol consumption. He had no history of glucocorticoid or antiviral medication use. The patient reported a recent history of a high-fat diet and a sedentary lifestyle. Regarding family history, his father and grandfather both had a history of diabetes mellitus. His father was slightly obese and had hypertriglyceridemia, with no history of coronary heart disease. His mother was healthy, with a normal body habitus, and denied any history of diabetes mellitus, hypercholesterolemia, hypertriglyceridemia, or coronary heart disease.

### Clinical findings

Upon admission, the patient exhibited stable vital signs: temperature 36.5 °C, pulse 78 beats/min, respiratory rate 18 breaths/min, and blood pressure 154/90 mmHg. Physical examination revealed: height 196 cm, weight 107.5 kg, body mass index (BMI) 27.98 kg/m², waist circumference 108 cm, hip circumference 110 cm. No other significant abnormalities were detected on physical examination.

### Diagnostic evaluation

**Figure 1:**
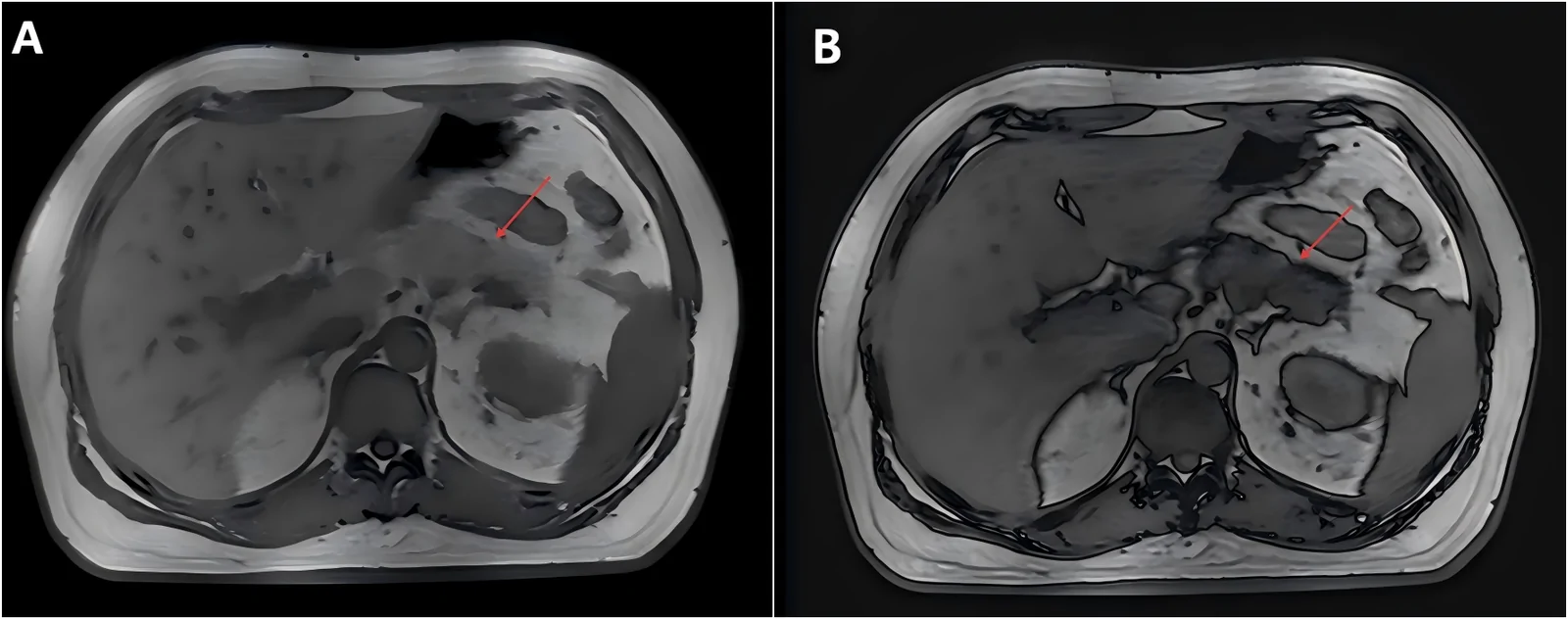
MRI of the patient's pancreas.

Comprehensive ancillary investigations were completed after admission; the patient’s admission laboratory results are shown in **Table 1**. Biochemical testing revealed significant abnormalities. Fasting blood glucose was 11.98 mmol/L and glycated hemoglobin (HbA1c) was markedly elevated at 10.5%. The lipid profile was severely deranged: triglycerides exceeded 37.87 mmol/L (diluted measurement: 91 mmol/L), total cholesterol was 20.49 mmol/L, and low-density lipoprotein cholesterol (LDL-C) was 5.37 mmol/L. Electrolyte testing revealed hyponatremia (sodium 124.7 mmol/L) and hypochloremia (chloride 90.7 mmol/L). Renal function tests showed creatinine 56.0 μmol/L and uric acid 63.8 μmol/L. Liver function, thyroid function, lipase, and amylase levels were within normal limits. Urinalysis revealed significant proteinuria: urinary microalbumin 564.6 mg/L, urinary microalbumin-to-creatinine ratio (UACR) 601.98 mg/g, and total urine protein 724 mg/L. The 24-hour urine protein excretion was 817.60 mg, with 24-hour urinary microalbumin 690.22 mg. Pancreatic autoantibodies, including anti-IA-2 antibody (anti-IA-2), glutamic acid decarboxylase antibody (GADA-65KD), insulin autoantibody (IAA), and islet cell antibody (ICA), were all negative. Pancreatic function assessment showed fasting C-peptide of 2.14 ng/mL and 2-hour postprandial C-peptide of 2.3 ng/mL, while proinsulin was markedly elevated at 490.3 pg/mL. Because long-term insulin therapy may affect serum insulin measurements, serum insulin was not measured directly. Instead, an indirect, non-standard estimate of the homeostasis model assessment of insulin resistance (HOMA-IR) of 7.82 was calculated from fasting C-peptide and fasting glucose, assuming a C-peptide-to-insulin molar ratio of 7:1 (7, 8). Pancreatic MRI revealed pancreatic fat infiltration (**Figure 1**: (A) T1WI in-phase: slightly increased signal intensity in the pancreatic parenchyma; (B) T1WI opposed-phase: unevenly decreased signal intensity in the pancreatic parenchyma). The following diagnoses were considered: type 2 diabetes mellitus, hypertriglyceridemia, insulin resistance, diabetic nephropathy, hepatic steatosis, and pancreatic steatosis.

**Table 1:** Laboratory test results of the patient upon initial admission.

Lab parameter	Value	Reference range
FBG	11.98	3.9-6.1 mmol/L
GLU-2h	14.91	
HbA1c	10.5	4.0-6.0%
TC	20.49	<5.18 mmol/L
TG	>37.87(after diluted: 91)	<1.70 mmol/L
HDL-C	1.75	>1.04 mmol/L
LDL-C	5.37	<3.37 mmol/L
ALT	7.0	9–50 U/L
AST	13.7	15–40 U/L
ALP	46.6	45–125 U/L
GGT	4.2	10–60 U/L
ALB	41.4	40–55 g/L
Cr	56.0	57-97 μmol/L
UA	63.8	208-428 μmol/L
AMY	24.3	<100 U/L
LIPA	11.8	13–60 U/L
Sodium	124.7	137–147 mmol/L
Chlorine	90.7	99–110 mmol/L
Fasting C-peptide	2.14	0.78-5.19 ng/mL
2-hour C-peptide	2.30	
Proinsulin	490.3	30.0-180.0 pg/mL
UACR	601.98	≤30 mg/g
TSH	2.97	0.56-5.91 mIU/L
FT3	4.99	3.53-7.37 pmol/L
FT4	12.64	7.98-16.02 pmol/L

FBG, fasting blood glucose; BG-2h, glucose-2 hours; HbA1c, glycated hemoglobin; TC, total cholesterol; TG, triglyceride; HDL-C, high density lipoprotein cholesterol; LDL-C, low density lipoprotein cholesterol; ALT, alanine aminotransferase; AST, aspartate aminotransferase; ALP, alkaline phosphatase; GGT, gamma-glutamyl transferase; ALB, albumin; Cr, creatinine; UA, uric acid; AMY, amylase; LIPA, lipase; UACR, urinary microalbumin-to-creatinine ratio; TSH, thyroid stimulating hormone; FT3, free triiodothyronine; FT4, free thyroxine.

To further elucidate the etiology of severe hypertriglyceridemia, targeted genetic testing was performed. No common pathogenic variants associated with familial chylomicronemia syndrome were identified in *LPL*, *APOC2*, *APOA5*, *GPIHBP1*, or *LMF1*. Unexpectedly, a heterozygous missense variant in the *LRP6* gene was identified: NM_002336.3: c.3107A>G (p.Asn1036Ser). This variant (rs143873199) is located at 12p13.2 and has an extremely low population frequency (gnomAD: 39/256, 019; allele frequency = 0.00015233). The variant was detected by targeted panel sequencing on the DNBSEQ-T7 platform and confirmed by Sanger sequencing. Unfortunately, the patient’s father was lost to follow-up, and the parental origin of the variant could not be determined. The genetic testing results and variant interpretation are presented in **Table 2**.

**Table 2:** Genetic testing results and variant interpretation of the patient.

Category	Details
Basic information and positioning	
Gene	LRP6
Transcript	NM_002336.3
Exon	Exon 14
Nucleotide amino acid	c.3107A>G(p.Asn1036Ser)
Variation type	Missense/Single Nucleotide Variation (SNV)
Heterozygosity	Heterozygous
Chromosome location	chr12:12301975
Cytogenetic bands	12p13.2
dbSNP ID	rs143873199
Population frequency and conservatism	
Group frequency (gnomAD v2.1.1)	0.00015233 (39/256, 019)
Conservatism (GERP++)	5.71 (highly conservative)
Pathogenicity calculation prediction (*in silica* pathogenicity)	
SIFT	0.037 (Damaging)
PolyPhen-2	0.631 (Possibly_damaging)
MutationTaster	1.0 (Disease_causing)
REVEL	0.491 (Uncertain)
AlphaMissense	0.1511 (Benign)
Splicing effect prediction	
SPIDEX	-0.0099 (no significant splicing effect)
dbscSNV	Not applicable (N/A)
spliceAI	Not applicable (N/A)
ACMG grading and evidence	
ACMG/AMP classification	Uncertain Significance - Based on PM2
Sequencing methodology and confirmatory testing	
Sequencing platform	DNBSEQ-T7
Sequencing type	Targeted gene panel sequencing
Confirmatory testing	Sanger sequencing

### Therapeutic interventions

**Figure 2:**
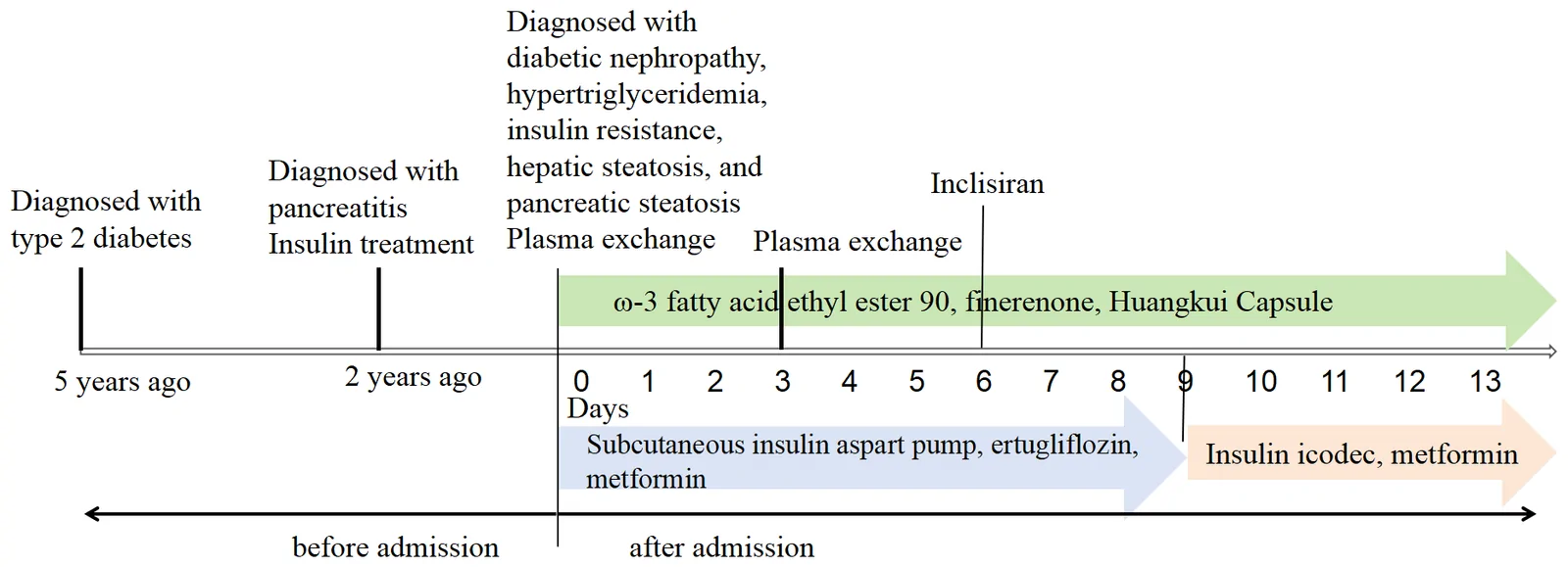
The timeline of the patient's clinical course and therapeutic interventions.

Given the patient’s markedly elevated triglyceride levels, plasma exchange was performed on the day of admission and again on hospital day 3 to rapidly reduce lipid levels. Concomitantly, ω-3 fatty acid ethyl ester 90 and subcutaneous inclisiran were administered for lipid-lowering therapy, together with a strict low-fat diet. For glycemic management, intensive glucose-lowering therapy was initiated with a subcutaneous insulin aspart pump (total daily dose, 50.2 units) combined with metformin and ertugliflozin. On day 9 of hospitalization, ertugliflozin was discontinued because of a genital tract infection. The regimen was subsequently adjusted to once-weekly subcutaneous insulin icodec at a dose of 190 units, combined with oral metformin 1g twice daily. For diabetic kidney disease, the nonsteroidal mineralocorticoid receptor antagonist finerenone and the traditional Chinese herbal formulation Huangkui capsules were used to reduce proteinuria. The patient’s clinical course and therapeutic interventions are presented in **Figure 2**.

### Follow-up and outcomes

**Figure 3:**
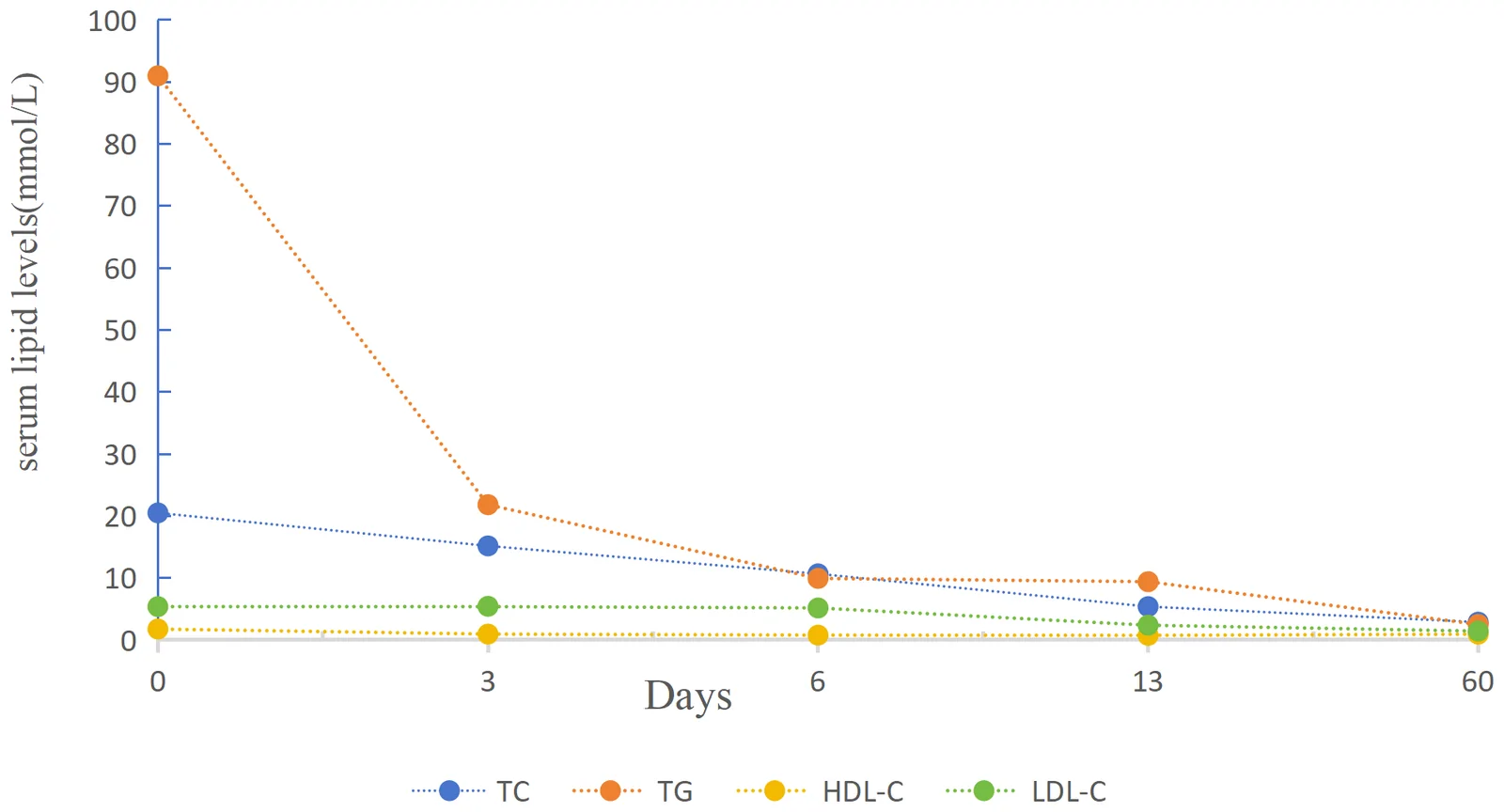
The changes in the patient's blood lipid levels.

After intensive multidisciplinary treatment, the patient’s lipid and glucose profiles improved substantially. On the third day after admission, repeat lipid testing showed a reduction in triglycerides to 21.81 mmol/L. By the sixth day of hospitalization, triglycerides had decreased further to 9.92 mmol/L, with total cholesterol 10.66 mmol/L and LDL-C 5.15 mmol/L. On the 13th day after admission, repeat testing indicated more favorable lipid control: triglycerides 9.39 mmol/L, total cholesterol 5.37 mmol/L, and LDL-C 2.37 mmol/L. At the two-month follow-up, lipid profiles were as follows: triglycerides 2.49 mmol/L, total cholesterol 2.87 mmol/L, and LDL-C 1.42 mmol/L. Changes in lipid levels are illustrated in **Figure 3**. Regarding glycemic control, after two weeks of inpatient treatment, fasting blood glucose was maintained at approximately 6 mmol/L, and 2-hour postprandial blood glucose was maintained at 8–10 mmol/L. At the two-month follow-up, HbA1c was 7%, and the UACR had decreased to 341.66 mg/g.

## Discussion

This case involved early-onset extreme hypertriglyceridemia (HTG), insulin resistance, pancreatic fat infiltration, and early-stage diabetic nephropathy. These clinical manifestations differed markedly from typical cases of hypertriglyceridemia-induced acute pancreatitis (HTG-AP) reported in the literature. The literature consistently indicates that HTG-AP typically occurs in individuals with pre-existing dysregulation of lipoprotein metabolism and is often precipitated by secondary factors such as uncontrolled diabetes and alcohol abuse; these patients generally exhibit greater disease severity and a higher risk of organ failure (9, 10). The present case is unusual in that the patient had extreme hypertriglyceridemia with a severe lipid phenotype coexisting with marked metabolic dysregulation and insulin resistance, manifested by abdominal obesity, hyperproinsulinemia, an estimated C-peptide–based insulin resistance index above 5, pancreatic fat infiltration, and hepatic steatosis. A comprehensive evaluation excluded common secondary causes of hypertriglyceridemia, including thyroid disease, drug-related factors, acute pancreatitis, familial chylomicronemia syndrome, and chronic renal failure, suggesting the possibility of an underlying etiology beyond the usual acquired factors. Although numerous studies have examined the association between obesity and type 2 diabetes mellitus (T2DM), most have focused on multifactorial insulin resistance and metabolic syndrome (11, 12). In the present case, the development of such pronounced insulin resistance and lipotoxicity at a relatively young age raises the possibility of an underlying genetic factor.

Comparison with known hereditary disorders of lipid metabolism makes the findings in this case particularly noteworthy. For example, familial chylomicronemia syndrome (FCS) and certain forms of congenital generalized lipodystrophy (such as Berardinelli-Seip syndrome) can present with severe hypertriglyceridemia, insulin resistance, and an increased risk of pancreatitis (13, 14). However, genetic testing in this case did not identify pathogenic variants associated with FCS but unexpectedly revealed a variant in the *LRP6* gene. The present case exhibited extreme hypertriglyceridemia, insulin resistance, hepatic steatosis, pancreatic steatosis, and diabetic nephropathy. In contrast, reviews of the genetic architecture of severe HTG indicate that most cases are polygenic or multifactorial, whereas reports implicating rare variants such as those in *LRP6* are extremely scarce (15). Thus, this case provides a clinical description of a patient carrying an *LRP6* variant of VUS and highlights the importance of comprehensive genetic evaluation in young patients with extreme metabolic abnormalities, to distinguish such cases from the more common FCS or multifactorial hypertriglyceridemia.

This case highlights the critical importance of a systematic diagnostic approach when young patients present with extreme and atypical metabolic phenotypes. Although the patient had a clear family history of diabetes, his clinical presentation could easily have been attributed to common type 2 diabetes combined with severe metabolic syndrome (16). However, features such as extreme hypertriglyceridemia, relatively low C-peptide levels disproportionate to the degree of hyperglycemia, and pancreatic fat infiltration collectively constituted a “red flag” that should prompt clinicians to consider an underlying genetic contribution (17). When confronted with such complex metabolic phenotypes, clinicians should not rely solely on a multifactorial explanation but should actively investigate potential genetic causes (18).

Genetic testing in this case helped exclude recognized monogenic causes of severe hypertriglyceridemia and identified an LRP6 VUS that warrants further investigation. In silico prediction tools yielded discordant results. Traditional algorithms, including SIFT, PolyPhen-2, and MutationTaster, predominantly predicted a deleterious effect, and the GERP++ score of 5.71 indicated high evolutionary conservation of the affected residue. However, the REVEL score was borderline (0.491), and AlphaMissense predicted the variant to be benign (score 0.1511). Because these computational predictions were discordant and did not provide a consistent consensus supporting pathogenicity, PP3 was not applied as evidence for pathogenicity. According to the ACMG/AMP guidelines, the variant was therefore classified as a variant of VUS; PM2 (extremely low frequency in population databases) was considered applicable. A causal relationship between this variant and the clinical presentation cannot be established at present; the findings point instead to a potential association that warrants further investigation. This raises a molecular hypothesis involving the LRP6–Wnt/β-catenin signaling pathway, which requires functional studies to clarify its potential role in this patient’s phenotype.

From a pathophysiological perspective, *LRP6* encodes low-density lipoprotein receptor-related protein 6, a key component of the Wnt/β -catenin signaling pathway that plays an important role in insulin signaling and lipid metabolism (19). Previous studies have shown that loss of *LRP6* function impairs downstream signal transduction, resulting in reduced insulin sensitivity and increased hepatic gluconeogenesis and lipogenesis (20, 21). In addition, lipotoxicity due to elevated free fatty acids—a consequence frequently associated with impaired insulin signaling and dyslipidemia—can directly impair pancreatic β-cell function, promote pancreatic steatosis, and potentially contribute to kidney injury and diabetic nephropathy. Together, these mechanisms provide a plausible biological framework linking LRP6/Wnt signaling dysfunction to the metabolic abnormalities observed in this patient.

Importantly, however, the evidence described above derives from general studies of the LRP6/Wnt signaling pathway rather than from functional characterization of the specific p.Asn1036Ser variant identified in this case. Whether this variant exerts any functional effect on *LRP6* activity—and consequently whether the proposed pathway is operative in this patient—remains entirely hypothetical and requires direct experimental validation. If future functional studies confirm that p.Asn1036Ser impairs LRP6-mediated Wnt signaling, the pathway outlined above could provide a mechanistic explanation for the observed phenotype. At present, however, this remains a research hypothesis to be tested rather than an established disease mechanism.

In summary, this case highlights that, in young patients presenting with extreme or unusual metabolic phenotypes, a structured and systematic diagnostic approach is critical for identifying the underlying etiology. Clinicians should recognize specific “red flags,” such as extreme HTG that is disproportionate to the degree of glycemic control, marked insulin resistance at a young age, and evidence of organ steatosis. These features should prompt a search for monogenic contributors beyond common type 2 diabetes (22). Although the *LRP6* variant of VUS identified in this patient is not conclusive evidence of pathogenicity, it provides a direction for future research.

Nevertheless, as a single-case report, this study has several limitations. First, the lack of functional validation of the *LRP6* variant and the absence of familial co-segregation analysis preclude the establishment of a causal relationship. The variant remains a VUS, and its precise role in the patient’s phenotype is speculative. Future studies should focus on functional characterization of the p.Asn1036Ser variant to clarify its impact on Wnt/β -catenin signaling and should screen for *LRP6* variants in larger cohorts of patients with insulin resistance and severe HTG. Such efforts will help elucidate the clinical spectrum of LRP6-related metabolic disorders.

## Data Availability

The raw data supporting the conclusions of this article will be made available by the authors, without undue reservation.
